# Shigellosis Linked to Sex Venues, Australia

**DOI:** 10.3201/eid0808.010534

**Published:** 2002-08

**Authors:** Belinda O'Sullivan, Valerie Delpech, Giulietta Pontivivo, Thomas Karagiannis, Debbie Marriott, John Harkness, Jeremy M. McAnulty

**Affiliations:** * New South Wales Health Department, New South Wales**,** Australia; † Prince of Wales Hospital, Sydney, Australia; and ‡St. Vincent’s Hospital, Sydney, Australia

**Keywords:** *Shigella*, outbreak, sex venues, homosexual

## Abstract

From January 1 to July 31, 2000, 148 cases of *Shigella* infection were reported in New South Wales, Australia, compared with an annual average of 95 cases. Of reported cases, 83% were confirmed as *Shigella* *sonnei* biotype G infections; 80% were in homosexual men. Visiting a sex venue in the 2 weeks before onset of illness was the only factor significantly associated with shigellosis.

In 2000, a major inner-city hospital laboratory in Sydney, New South Wales (NSW), reported to local health authorities an unexpected increase in the incidence of shigellosis in homosexual men. Shigellosis outbreaks have commonly been reported related to person-to-person contact (1), child-care centers, food sources (2), institutionalized populations (3), and contaminated water (4). The infectious dose is low, with 10–100 organisms/mL sufficient for infection (5). In the United States, reports in the 1970s linked shigellosis transmission to orogenital and oral-anal sexual contact between men in bathhouses (6–7) and more recently with underlying HIV infection (8). Recent clusters of *Shigella sonnei* infection have been identified in Canada (9) and San Francisco (10) in men who have sex with men.

Sex venues in Australia are commercial establishments or bathhouses where men pay an entry fee to engage in casual sex with other men. Such establishments may provide bondage equipment, cubicles for anonymous sex, saunas, lounges, douching facilities, and toilets. At the time of the outbreak, no guidelines governed infection control in these venues.

## The Study

We contacted all public and private microbiology laboratories in inner Sydney as well as state and national reference laboratories to identify cases of shigellosis in NSW and determine the average number of cases per year. We defined outbreak-associated cases as shigellosis in homosexual men resident in NSW, aged 19–66 years, and identified by laboratories to have *Shigella* *sonnei* biotype G (SSBG) infection or untyped *S. sonnei* (if the laboratory did not routinely biotype *S. sonnei*) from April 1 to July 31, 2000. A physician-administered questionnaire, piloted in 1999, included demographic details and history of illness, sexual activity, dining out, and overseas travel. Physicians from five key medical centers in inner Sydney specializing in homosexual men's health agreed to seek verbal consent from patients to either complete the questionnaire or be contacted by the investigators by telephone.

We compared reported risk exposures of patients with controls who completed the same questionnaire (all physician-administered) at the same medical centers from March 1 to July 31, 1999 (Delpech, unpub. data). Controls were defined as homosexual or bisexual male residents of NSW who did not report any diarrhea in the previous 3 months.

We contacted all sex venues in inner Sydney by telephone to request permission to conduct an audit of hygiene and infection control practices. An infection control nurse inspected each venue, completing a standard audit tool that covered the appropriateness of lighting and surfaces for cleaning, cleaning regimens, hand washing, douching facilities, condom availability, and staff education. Microbiologic swabs were taken from contact surfaces including mattresses, cubicle walls, bondage equipment, door handles, and lubricant dispensers and placed in transport medium.

Aerobic cultures were performed on blood agar and MacConkey agar plates. Organisms were identified on Gram stain and routine biochemical testing. Antimicrobial susceptibility was performed by the National Committee of Clinical Laboratory Standards method with ciprofloxacin, co-trimoxazole, ampicillin, and cefotaxime. Clonality was demonstrated by using pulsed-field gel electrophoresis, enterobacterial repetitive intergenic consensus, and random-amplified polymorphic DNA polymerase chain reaction.

Univariate and multivariate logistic regression analysis was conducted by using Statistical Analytic Software (SAS; SAS Institute Inc., Cary, NC). Variables with p values <0.25 were applied to the multivariate model initially, and the backward stepwise elimination method was used. “Casual sexual partners” were defined by reporting “having casual sex partners in the last 3 months.”

One hundred forty-eight patients with *Shigella* infection were identified from January 1 to July 31, 2000, in NSW; 123 (83%) were confirmed as having SSBG infections, compared with an annual average of 95 cases, with about 50% typed as SSBG (11). Most of the patients were reported during April and May (N=89) (Figure). Of the 123 patients with confirmed SSBG, 98 were identified as homosexual men ages 16–66 years and were defined as outbreak-associated cases. Of these, 15 (15%) were excluded because they had no physician-contact details, as these details were not routinely collected by state and national reference laboratories.

**Figure:**
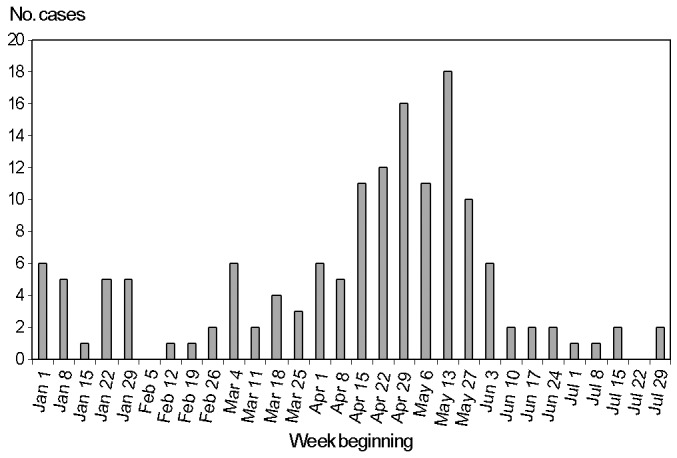
*Shigella sonnei* biotype G laboratory reports, by date of specimen collection, New South Wales, January 1–July 31, 2000.

Questionnaires were completed by 42 (51%) of the remaining 83 patients; 33% of 42 questionnaires were completed by telephone interview with the investigators. Similar proportions of patients who completed a questionnaire (N=42) lived in inner Sydney (64%) compared with all outbreak-associated cases (N=148) (58%); both groups had a median age of 38 years. The main reason questionnaires were not completed was refusal to participate.

All 42 patients reported diarrhea, 19 (45%) bloody diarrhea, 35 (83%) fever, 38 (90%) cramps, and 12 (29%) vomiting. Illness lasted a mean of 13 days (range 2–90 days), and eight (19%) were hospitalized. Twenty-two (52%) reported that they were HIV infected. HIV-infected patients were no more likely to be hospitalized (odds ratio [OR] 0.9; 95% confidence interval [CI] 0.2 to 5.3).

Sixty-five controls were included in the case-control study. Controls had a mean age of 38 years. Sixty-four percent of patients and 55% of controls resided in inner Sydney (OR 1.5; 95% CI 0.6 to 3.5). More patients (52%) than controls (48%) reported that they were infected with HIV (OR 1.2; 95% CI 0.6 to 2.6). Patients who reported HIV infections were significantly more likely to report diarrhea than those not reporting HIV infection (OR 2.9; 95% CI 1.5 to 5.9).

In univariate analysis, patients were more likely than controls to report having casual sex partners; visiting a sex venue in the previous 3 months; visiting a sex venue and having more than one sex partner in previous 2 weeks; and dining out in the last 3 days (Table). Age, either expressed as a continuous variable or in categorical 5-year age groups (OR 1.0; 95% CI 1.0 to 1.1); not always washing hands after sex in the previous 2 weeks; traveling overseas; and specific sexual behaviors or HIV status were not associated with illness. In multivariate analysis, visiting a sex venue in the previous 2 weeks was the only significant independent risk factor for shigellosis (p=0.002; OR 4.8; 95% CI 1.8 to 12.6).

**Table:** Characteristics of shigellosis patients and controls, New South Wales, Australia, April 1– July 31, 2000

	Patients N=42 (%)	Controls N=65 (%)	Crude odds ratio (95% CI^a^)
Casual sex partners in the last 3 months	37 (88)	46 (71)	3.1 (1.0 to 9.0)^b^
Visited a sex venue in the last 3 months	31 (74)	28 (43)	3.6 (1.6 to 8.5)^b^
Visited a sex venue in the last 2 weeks	24 (57)	14 (22)	4.8 (2.1 to 11.4)^b^
More than one sex partner in the last 2 weeks	21 (50)	21 (32)	3.1 (1.3 to 7.5)^b^
Any sex in the 2 weeks before onset of illness^c^	37 (88)	52 (80)	Incalc^d^
Oral receptive sex in the last 2 weeks^d^	35 (83)	48 (74)	1.77 (0.7 to 4.7)
Anal insertive sex in the last 2 weeks^d^	26 (62)	32 (49)	1.7 (0.8 to 3.7)
Anal receptive sex in the last 2 weeks^d^	25 (60)	28 (43)	1.9 ( 0.9 to 4.3)
Oral-anal insertive sex in the last 2 weeks^d^	13 (31)	22 (34)	0.9 (0.4 to 2.0)
Digital insertive sex in the last 2 weeks^d^	26 (62)	31 (48)	1.8 (0.8 to 3.9)
Not always washing hands after sex in the last 2 weeks	16 (38)	19 (29)	1.4 (0.6 to 3.2)
Dined out at a commercial food outlet^c^ in the last 3 days before onset of illness	25 (60)	27 (42)	2.5 (1.1 to 5.8)^b^
Traveled overseas in the last 3 months	9 (24)	12 (18)	1.3 (0.5 to 3.4)
HIV positive	22 (52)	31 (48)	1.2 (0.6 to 2.6)

^a^90% CI, 95% confidence intervals. ^b^Significant at p<0.05 ^c^For controls, this question was asked in relation to previous 2 weeks rather than the 2 weeks before onset of illness. ^d^Missing values were excluded from the analysis except for sexual activity variables (e.g., oral insertive sex), for which participants were asked to indicate “yes” if they did the specified activity. As such, failure to answer these questions was considered a “no” response.

All 15 identified sex-venue chains in inner Sydney were inspected during July 1 through August 1, 2000. Six (40%) of 15 had dim lighting that would prevent adequate cleaning. Ten (67%) had inadequate cleaning products and surfaces that were in a state of disrepair, including chipped, cracked, or damaged floors, wall surfaces, and furniture. Only four (27%) had a routine cleaning regimen during operational hours.

Six (40%) sex venues had no hand-washing basins, and two (13%) had basins that were inaccessible to patrons in sex activity areas. Five (33%) had anal-douching facilities, all of which sold douching tubing that was not designed for anal insertion. Only two venues with douching facilities reported routine cleaning of douching facilities after use. In one venue, fecally contaminated douching tubing was found stored in the douching room, suggesting re-use.

Seven (47%) venues offered unlimited access to condoms and lubricants, and eight (47%) dispensed one condom and lubricant sachet on entry. Only two venues reported that staff members received infection control education.

A total of 63 microbiologic swabs were taken from 11 venues. No *Shigella* species were isolated, but 18 (29%) cultures, including 6 of 12 sites from one venue, grew coliform bacteria, indicating fecal contamination. Eight (57%) of 14 mattress swabs from different venues grew coliforms. Environmental organisms were isolated from 36 (57%) swabs. Ninety percent of case isolates were resistant to ampicillin and cotrimoxazole, and 98% showed a similar pattern of clonality.

Given the variability of standard infection-control practices across sex venues, *Shigella* may have been transmitted either directly during casual sex or indirectly from contact with contaminated surfaces or douching equipment. While visiting a sex venue was the only significant risk factor associated with shigellosis, 40% of patients reported not having attended a sex venue. Other factors that we did not measure may have led to transmission in these persons, including casual or sexual contact with other people with shigellosis outside sex venues, contact with fomites, or eating contaminated food. The foodborne route is unlikely, as this outbreak did not affect the general community.

Despite active surveillance, some underreporting of cases is likely in this outbreak because not all patients would have consulted a physician or had a fecal specimen obtained. However, the rate of physician visits for shigellosis is likely to be higher than for other less severe diarrheal illnesses (12). While the use of historical controls makes evaluating food- and waterborne risk factors for shigellosis difficult, we believe that the evaluation of sexual behaviors in homosexuals is likely to be reliable as they show little variation over the study period (13).

An interagency approach was used to develop and conduct plans to control the outbreak. Actions included a health promotion campaign focused on homosexual men; a shigellosis forum attended by owners, managers, and cleaners of sex venues; and the interagency development of infection control guidelines for such establishments. Guidelines for infection control should be followed and equipment and surfaces in sex venues should be cleaned regularly in adequate lighting. Patrons should have easy access to and be encouraged to use hand-washing facilities to minimize the likelihood of transmission of enteric pathogens. Homosexual men should routinely be given information about the ongoing risk of transmission of enteric pathogens.
